# Photoconvertible Fluorescent Proteins and the Role of Dynamics in Protein Evolution

**DOI:** 10.3390/ijms18081792

**Published:** 2017-08-18

**Authors:** Rebekka M. Wachter

**Affiliations:** School of Molecular Sciences and Center for Bioenergy and Photosynthesis, Arizona State University, Tempe, AZ 85287, USA; rwachter@asu.edu; Tel.: +1-480-965-8188

**Keywords:** green fluorescent protein, proton transfer, reverse protonation

## Abstract

Photoconvertible fluorescent proteins (pcFPs) constitute a large group of fluorescent proteins related to green fluorescent protein (GFP) that, when exposed to blue light, bear the capability of irreversibly switching their emission color from green to red. Not surprisingly, this fascinating class of FPs has found numerous applications, in particular for the visualization of biological processes. A detailed understanding of the photoconversion mechanism appears indispensable in the design of improved variants for applications such as super-resolution imaging. In this article, recent work is reviewed that involves using pcFPs as a model system for studying protein dynamics. Evidence has been provided that the evolution of pcFPs from a green ancestor involved the natural selection for altered dynamical features of the beta-barrel fold. It appears that photoconversion may be the outcome of a long-range positional shift of a fold-anchoring region. A relatively stiff, rigid element appears to have migrated away from the chromophore-bearing section to the opposite edge of the barrel, thereby endowing pcFPs with increased active site flexibility while keeping the fold intact. In this way, the stage was set for the coupling of light absorption with subsequent chemical transformations. The emerging mechanistic model suggests that highly specific dynamic motions are linked to key chemical steps, preparing the system for a concerted deprotonation and β-elimination reaction that enlarges the chromophore’s π-conjugation to generate red color.

## 1. Introduction

**Figure 1:**
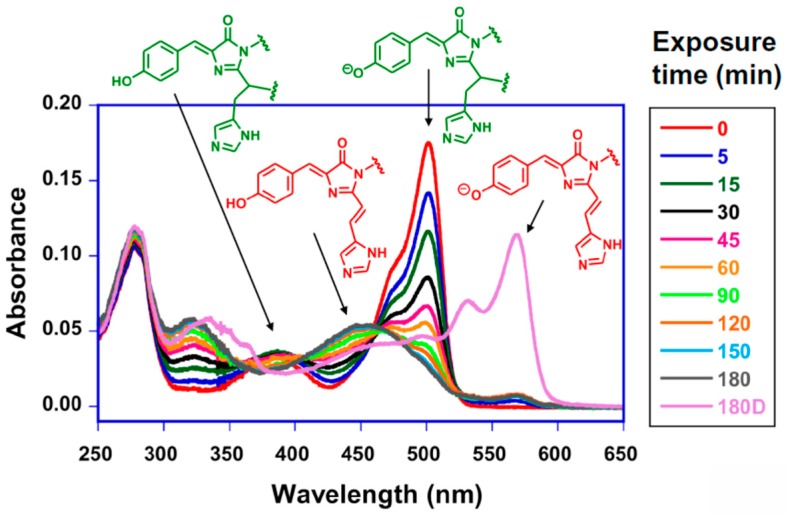
Timecourse of photoconversion of the pcFP least evolved ancestor (LEA). Experiments were carried out at pH 5.7 with 405 nm LED illumination [13]. The exposure times (0 to 180 min) are listed in the box. After 180 min exposure, the sample was dark-adapted overnight (180D), providing evidence for a slow conformational relaxation step that favors the anionic chromophore. The respective maxima for green and red chromophores in their neutral and anionic states are indicated, and the chemical structures of these chromophores are shown in green and red.

In green fluorescent protein (GFP)-like proteins, chromophore dynamics has been linked to fluorescence quantum yield [1], excited-state proton transfer [2], reversible dark state formation [3,4], and addition/elimination reactions [5]. Maybe not surprisingly, evidence has been accumulating that the green-to-red photoconversion process in photoconvertible fluorescent proteins (pcFPs) is linked to chromophore dynamics as well [6,7]. pcFPs are a class of GFP-like proteins able to undergo a light-dependent irreversible chemical conversion that generates a three-ring chromophore from the two-ring GFP-like chromophore [8]. The exposure to UV or violet radiation changes the emission from about 518 to about 582 nm (Figure 1), a feature that was first described for the pcFPs Kaede [9] and EosFP [10]. Although many extant variants with this characteristic have since been isolated from reef-building corals, and numerous others have been generated by genetic engineering methods [11], deciphering the catalytic mechanism of light-triggered color conversion has posed substantial challenges. In large part, this appears to be due to the difficulty of generating sufficient populations of relevant intermediate forms [8,12,13]. A particular challenge originates from experimental limitations in capturing relevant structural motions on intrinsic molecular timescales [14,15].

## 2. General Characterization of Photoconvertible Fluorescent Proteins (pcFPs)

**Scheme 1:**
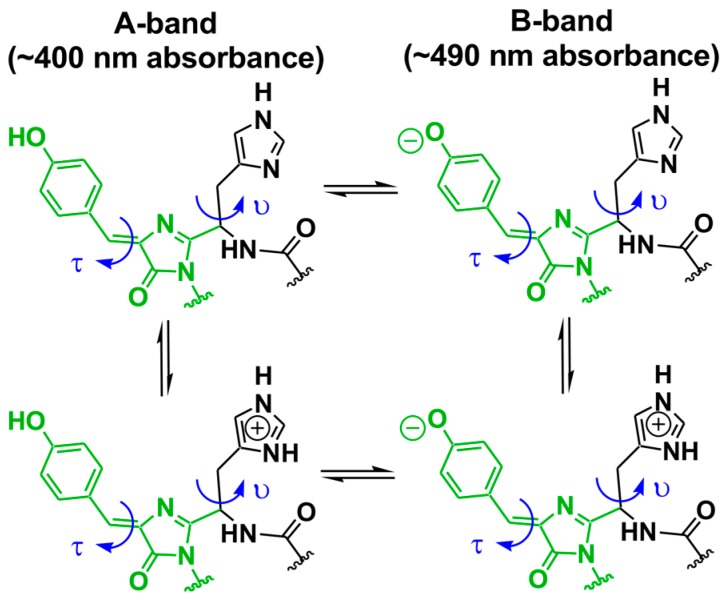
Bond rotations proposed to be critical in light-activated photoconversion of photoconvertible fluorescent proteins (pcFPs) [13]. The rotation angles τ (chromophoric β-methylene bridge) and υ (His62 side chain) are highlighted.

**Scheme 2:**
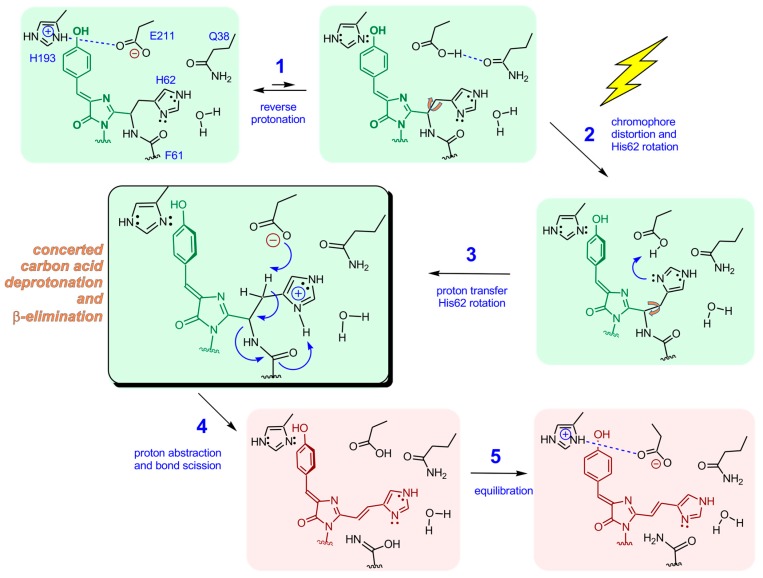
Proposed photoconversion mechanism in pcFPs according to Kim et al., 2015 [13]. The yellow arrow indicates light exposure. Reverse protonation is thought to promote activation of Glu211 and His62 by allowing chromophore distortions.

In all reported cases, green-to-red pcFPs contain the sequence His-Tyr-Gly buried in the interior of the eleven-stranded β-barrel. Frequently, the residue numbers of this tripeptide are 62–64, a numbering system that will be used here. However, loop insertions and N-terminal extensions may modify the corresponding residue numbers somewhat, as in avGFP (Aequorea victoria green fluorescent protein), where the equivalent residues are 65–67. The first step in pcFP maturation is the spontaneous formation of a green chromophore identical to that found in avGFP. This process requires properly folded protein and involves an internal main-chain cyclization reaction, followed first by a two-electron oxidation where molecular oxygen serves as electron acceptor, and subsequently by the elimination of water [16,17]. Once this process is complete, the absorption of UV or blue light by the protonated neutral form of the green chromophore (state A, Scheme 1), which is in equilibrium with its anionic state (state B, Scheme 1) [10], triggers the incorporation of the His62 side chain into the chromophore’s π-overlap system [18]. In this way, a three-ring chromophore is generated that entails a desaturated His62 C_α_–C_β_ bond, with concomitant red-shifting of absorbance and fluorescence spectra. This process requires main-chain bond scission that involves a β-elimination reaction, in which the His62 amide nitrogen is ejected from the His62 α-carbon (Scheme 2). In general, pcFPs remain natively folded upon photoconversion, in spite of the interior backbone cleavage that provides an N-terminal 10 kDa and C-terminal 18 kDa peptide fragment upon protein denaturation. Several studies have indicated that pcFP red chromophore formation is not likely to involve excited-state proton transfer reactions (ESPT) or one-electron transfer reactions, as intermediates consistent with such processes were not detected [19,20].

## 3. Least Evolved Ancestor (LEA) Series of pcFPs Developed by Ancestral Gene Reconstruction Technology

Some years ago, gene reconstruction technology was utilized, in part to gain a better understanding of the catalytic mechanism operational in pcFPs [21,22]. A key advantage of the ancestral reconstruction approach is the ability to study photoconversion in the absence of any ballast mutations that may be due to genetic drift or natural selection for unrelated adaptations. A series of sequences were predicted that were judged to be consistent with the common ancestor of all color classes expressed in the extant great star coral *Monastrea cavernosa* (cyan, green and red). These posterior predictions formed the basis for the experimental construction of a large set of ancestral proteins via combinatorial methods [22]. As these sequences encoded green-fluorescent proteins only, the ancestral node delineated by these sequences was termed ALL-GFP. The pair of ancestral green and extant photoconvertible proteins most similar to each other entailed a total of 37 amino acid substitutions, allowing for the experimental testing of each of these sites for its involvement in the generation of red color. Statistical data analysis provided a direct link to pcFP evolution for only 13 out of the 37 sites. The subsequent production of several possible evolutionary intermediates along this lineage nicely illuminated phenotypic change along clade D of the stony corals (order *Scleractinia*, suborder *Faviina*) [21,23,24].

**Figure 2:**
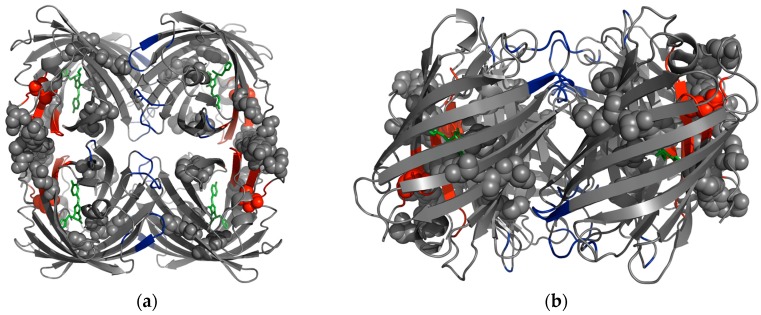
Tetrameric X-ray structure of the common green ancestor bearing the Q62H substitution, ALL-Q62H [13]. Residues that were mutated to generate the pcFP LEA are shown as spheres (see Table 1). Residues with large changes in percent differential flexibility index (∆*dfi*) values (top 15%) are shown in red (large increase in flexibility, residues 67–71, 142, 187–193, 213–215) and blue (large decrease in flexibility, residues 19–22, 50, 97–98, 125, 127–129, 165, 167). The chromophore is shown in green. (**a**) Top view; (**b**) Side view.

Proteins generated in this way included the common green ancestor ALL-GFP and its single-substitution variant ALL-Q62H, which bears the functionally important His residue in position 62. Neither of these ancestral proteins are able to undergo the photoconversion process, and are therefore non-pcFPs. Subsequently, ALL-Q62H was chosen as background for the step-wise introduction of additional functionally relevant substitutions. The partially evolved intermediate variants derived from ALL-Q62H were termed LEAX6, LEAX72 and LEAX121, with each exhibiting only weak pcFP activity. Finally, a protein termed the Least Evolved Ancestor (LEA) was produced, a variant that bears a total of 13 mutations compared to ALL-GFP (Table 1 and Figure 2). This set of mutations was judged to be the minimum number of mutations both necessary and sufficient to generate a red-fluorescent chromophore upon light excitation, as LEA displayed high photoconversion efficiency [22].

In subsequent work, we demonstrated that the LEA quantum yield of photoconversion is 1.5 × 10^−3^ [25], a value that is similar to other well-characterized pcFPs. Other experiments demonstrated that among the 13 sites, the substitutions T69A and Y116N and the deletion of Tyr217 were particularly important in promoting red color [22]. With the exception of Q62H, which introduces the indispensable imidazole building block, all single-site reversions retained their ability to photoconvert, albeit with reduced efficiency. Although within the series of ancestral reconstructed proteins, the tetrameric assembly of the common green ancestor ALL-GFP was shown to remain intact [13], it is clear that the maintenance of quaternary structure is not a prerequisite for photoconversion [26,27].

## 4. The pcFP LEA Is More Dynamic than Its Non-pcFP Precursor Protein

A few years ago, we carried out a detailed biophysical characterization of the ancestral series of variants described above [13,25]. Information derived from atomic resolution X-ray crystal structures of the green (pre-photoconversion) state of several variants was combined with computational approaches to elucidate the evolutionary role of local and global protein dynamics in the natural selection for photoconversion. Equilibrium molecular dynamics (MD) simulations of the ALL-Q62H and LEA tetrameric β-barrel assemblies provided evidence for increased chromophore dynamics in LEA compared to its precursor protein. In these computations, the α-carbon fluctuations of LEA were significantly higher than those of ALL-Q62H over a trajectory of 220 ns [13]. In addition, a significant reduction in thermostability was observed for the evolved, photoconversion-competent variants [13]. Although the α-carbons of the crystallographic models of LEA and ALL-Q62H were superimposable within error, and the positions of all catalytic groups were well-conserved, the MD and thermostability data suggested that the backbone of LEA undergoes an increased range of thermal motion compared to ALL-Q62H, and that its chromophore is more flexible.

## 5. pcFPs Harbor a Softer, More Dynamic Active Site and a Remote Knob-Like Region That Is Highly Rigidified

To aid in identifying specific regions of LEA that have incurred substantial changes in flexibility during the course of evolution from a green ancestor, we employed a computational method termed perturbation response scanning (PRS) [28,29]. Using this method, we calculated a residue-by-residue dynamic flexibility index (*dfi*) [13]. According to the canonical PRS model, the protein is typically viewed as an elastic network (Elastic Network Model or ENM), in which each node represents one residue and harmonic interactions occur between pairs of nodes. However, to apply PRS to LEA and ALL-Q62H, proteins with different residue identities at some positions, we used all-atom MD simulations rather than a coarse-grained network model in the PRS calculations. The incorporation of equilibrated MD trajectories allows for this approach to take non-covalent interactions into account.

In PRS, a random unit force or perturbation is applied sequentially to individual residues. The Brownian kick then travels through the residue interaction network, and induces various degrees of positional displacement in each of the remaining residues. The displacement of each residue *i* in the protein chain is recorded upon perturbation of residue *j*, and the process is repeated until all residues have received a Brownian kick one-by-one. To compute the residue-specific *dfi* value, the average displacement of a particular residue from its equilibrium position is calculated and normalized with respect to the average displacement of all sites. In this way, one can determine whether the average displacement of residue *i* is above or below the average displacements of all other residues, thereby allowing for the identification of particularly dynamical and particularly rigid sites in a protein chain.

Using this method, we made the surprising observation that ALL-Q62H and LEA exhibited large differences in flexibility for two diagonally opposite sections of the beta barrel, where elevated and depressed %*dfi* values (normalized *dfi* values) seemed to have switched place during the course of evolution (Figure 2). It is particularly noteworthy that residues with large differences in %*dfi* values (i.e., large ∆*dfi* values, such as the top 15% colored blue and red in Figure 2) do not involve mutational sites, with the sole exception of residue 69 (Table 1). This observation suggests that most of the substituted positions transmit perturbations through the protein matrix to other, more remote residues, rather than undergoing large changes in flexibility themselves. As an aside, the calculated differences ∆*dfi* between ALL-Q62H and LEA did not follow the same trend as the observed differences in (normalized) crystallographic B-factors [13]. This is not surprising, given that crystallographic B-factors reflect a variety of physical mechanisms related to disorder in the crystal.

Based on the ∆*dfi* analysis, it appears that LEA carries a substantially softer, more malleable active-site that is somehow linked by the protein matrix to the opposite corner of the β-barrel, which has acquired enhanced rigidity. This knob-like region could be considered a hinge or anchoring region for global protein breathing motions that ensures that the protein remains folded. As backbone shifts were essentially absent in the X-ray structures [13], the computational results nicely illustrate the impact of protein dynamics on phenotypic change. The emerging model suggests that during the course of evolution, the hinge region controlling vital low-frequency normal modes has migrated across the barrel, thereby setting the stage for phenotypic change. This model supports the notion that pcFPs require a more dynamic active site that allows for more substantial light-induced chromophore movements, as well as a histidine-mediated proton shuttling mechanism, as described in more detail below (Figure 2).

## 6. The Charge States of Buried Functional Groups Appear to Control the Rate of Color Change

Several years ago, we carried out extensive photoconversion kinetics on the pcFP LEA [25]. The results demonstrated that the photoconversion rate decreases both with increasing acidity and with increasing basicity. The bell-shaped pH-rate profile was fit to appropriate kinetic models to estimate the apparent apparent acid dissociation constant (pK_a_^app^) values that control the observed rates [25]. In the vicinity of the chromophore, a set of four titratable side chains, Glu211, His193, Glu144 and Arg66, form a quadrupolar arrangement of tightly coupled alternating charges. Therefore, the observed pK_a_^app^ values were attributed to Glu211 in combination with Glu144 for the up-slope (pK_a_^app^ = 4.5), and to His193 (pK_a_^app^ = 7.5) for the down-slope. According to this interpretation, the carboxylates of the two glutamic acids are primarily negatively charged, and His193 and Arg66 are primarily positively charged between pH 4.5 and 7.5, which is the pH region with highest photoconversion rates. Interestingly, the pK_a_ value of 6.3 of the LEA chromophore itself lies in this region as well, consistent with the notion that photoexcitation of the neutral form may be responsible for the ensuing photochemistry.

## 7. Transient Reverse Protonation of His193-Glu211 May Facilitate Deformation of the Chromophore in the Electronically Excited State

Over the years, numerous comparisons of X-ray structures of green and red pcFPs have demonstrated that photoconversion does not lead to structural alterations, with the sole exception of main-chain bond scission [8]. The Glu211 side chain is thought to provide the catalytic base for proton abstraction from His62-C_β_ (Scheme 2). The resulting desaturation of the His62 side chain provides a connection of the imidazole π-system to the π-system of the green chromophore, and therefore leads to a red-shifted spectrum [19,30]. For Glu211 to function as a strong base, the salt bridge to His193 must be temporarily disrupted, a process that may be set in motion by twisting of the chromophore around its β-methylene bridge. An increase in the spatial separation of His193 and Glu211 would be substantially facilitated in the reverse-protonated state, likely the catalytically competent charge state (Scheme 2, steps 1 and 2) [25]. The pH-rate profile suggests that at its apex, about 0.1% (calculated using the expression 10^−ΔpKa^) of all protein chains would bear neutral His193 and Glu211 side chains [31] (Scheme 2, step 1). It is worth noting that the photoconversion quantum yield, i.e., the fraction of absorbed photons that yields a red chromophore, is of similar order of magnitude to those reported for a variety of different pcFPs with values ranging from 10^−3^ to 10^−4^ [9,13,32].

Chromophore twisting in fluorescent proteins has been studied extensively by several research groups, and has been shown to bear the potential to substantially remodel the active site. For example, photoswitchable FPs such as monomeric teal-fluorescent protein mTFP and Dronpa exemplify proteins that are not able to convert their chromophores from green to red, yet do undergo light-dependent *cis*-*trans* isomerization reactions [3,4,33,34]. Recently, Dronpa chromophore isomerization was shown to occur on a time scale of 9 ps, and the quantum yield for photoswitching was shown to be only 0.00032 [35]. This value is not much different from the photoconversion quantum yield observed in pcFPs. In photoswitchable FPs, the dark state bears a distorted chromophore in the *trans* conformation that is accommodated by structural rearrangements of nearby residues [3]. These can only occur if the His193-Glu211 salt bridge is temporarily broken, a critical observation that provides additional support for the reverse protonation model (Scheme 2). Interestingly, photoswitching FPs appear to have evolved along the same evolutionary branch as photoconversion, and several pcFPs have been demonstrated to undergo significant photoswitching events as well [6].

## 8. Functional Group Activation and Active Site Geometry Support a Concerted, One-Step Proton Abstraction and β-Elimination Reaction

Recently, we have proposed that upon photoexcitation, the restructuring of the active site facilitates a side-chain rotamer adjustment of His62 (Scheme 1 and Scheme 2 and step 3) [13]. Bond rotation would place the imidazole N_δ1_ near the carboxylic acid of Glu211 in a geometry conducive to proton transfer to yield the imidazolium cation. In the ground state at equilibrium, His62 is judged to be neutral over a broad pH range, as it is positioned in a relatively hydrophobic environment without obvious hydrogen bonding interactions [8]. However, His62 imidazole protonation by Glu211 would activate its β-carbon for subsequent proton removal by Glu211, which now carries a negative charge that lacks stabilizing interactions (Scheme 2, step 4). Proton transfer from the His62 imidazolium N_δ2_ to the amide leaving group may occur as part of the same step, thereby preventing the formation of a carbanion at His62 C_β_ (Scheme 2, step 4). Therefore, we have proposed that the backbone cleavage step may be of concerted nature [25], reminiscent of another concerted mechanism proposed more than a decade ago [19]. Interestingly, crystal structures of a highly engineered pcFP termed KikGRX, a crystallizable variant of KikGR, have demonstrated that, in some cases, illumination can generate His62 side-chain conformers that would facilitate proton abstraction from Glu211 [36]. Although these X-ray structures represent equilibrium states rather than transient processes, they nicely demonstrate that the active site is flexible enough to allow for His62 rotamer adjustments. Therefore, a short-lived state entailing a highly basic Glu211 carboxylate and an acidified His62 β-carbon seems reasonable, and would prime the system for β-elimination (Scheme 2, step 4), directly yielding a three-ring chromophore with red-shifted optical properties (Scheme 2, step 5).

## 9. LEA Is Photoconversion-Competent Because of Functional, Epistatic and Compensatory Substitutions

In combination, the set of mutations introduced into ALL-GFP to generate LEA (Table 1) provides the necessary structural and dynamical framework for light-induced catalysis by the protein fold. These substitutions may primarily be functional in nature, such as Q62H, which becomes incorporated into the red chromophore. Other substitutions may primarily serve an epistatic role, such as the strategic replacement of some larger residues with smaller ones to provide room for chromophore twisting and His62 side-chain rotations (Scheme 1), as well as to ensure a productive directionality for such movements. Other epistatic mutations may serve to anchor the chromophore’s imidazolinone ring, or to dislodge the C-terminus to loosen up the chromophore-bearing pocket. A third group may be called compensatory by nature, as these substitutions are further removed from the chromophore, yet are absolutely critical for thermal stabilization. In this way, compensatory mutations allow for increased plasticity of the active site by allosterically mediating the formation of a rigid knob remote from the chromophore.

## 10. Concluding Remarks

### 10.1. Protein Dynamics and Protein Evolution

Protein motions occur on multiple timescales, ranging from fast local dynamics, such as atomic fluctuations and side-chain rotations within an enzyme’s active site, to slower collective movements that are allosterically propagated through networks of residues. Main-chain and side-chain dynamics have been shown to play significant roles in a broad variety of enzymatic transformations. Conformational fluctuations are thought to be tightly linked to a protein’s biological function, and may directly impact specific microscopic steps in enzyme catalysis [37,38]. Notably, dynamics has been shown to play a critical role in the evolution of proteins towards new functionalities [39,40,41,42,43]. In light of this notion, the significance of the ancestral pcFP reconstruction work discussed here [13] lies in the demonstration that phenotypic change along a branch of the protein family tree may come about solely by changes in local and collective motions, without any structural changes of equilibrium positions. Therefore, modifications in tertiary or quaternary structure, or positional shifts of catalytic groups, do not appear to be required for the evolution of new function.

### 10.2. Design of Improved pcFPs for Super-Resolution Microscopy

pcFPs are of strategic importance in the advancement of super-resolution imaging by localization microscopy and provide the basis for a myriad of applications in cell biology [44,45,46,47]. We hope that a more complete understanding of the mechanism of photoconversion in relation to structural dynamics will help extract rational design principles that can be exploited to address some of the shortcomings of currently available pcFPs. As a cell biological tool, the development of pcFPs with highly efficient conversion rates upon excitation at longer wavelengths would be highly desirable.

## Figures and Tables

**Table 1:** Differences in amino acid residues between the common green ancestor ALL-GFP and least evolved ancestor (LEA) [21,22]. The changes in percent *dfi* values (∆*dfi*, range −0.5 to +0.5) and corresponding aequorea victoria green fluorescent protein (avGFP) residue numbers are also provided [13].

Residue No.	ALL-GFP	LEA	∆*dfi*	avGFP Residue No.
60	A	V	−0.03	63
62	Q	H	+0.23	65
69	T	A	+0.46	72
74	D	H	−0.04	77
104	T	R	−0.17	109
105	S	N	+0.09	110
116	Y	N	+0.05	121
154	M	T	−0.04	162
157	V	I	−0.06	165
194	R	C	+0.23	204
216	R	H	+0.15	227
217	S	(deleted)	n/a	228 *
219 (218 for LEA)	L	G	n/a	229 *

n/a: not applicable; * The alignment of the avGFP residues 228 to 232 with the C-termini of the synthetic constructs is not reliable.

## Abbreviations

ALL-GFP	Common green ancestor
LEA	Least evolved ancestor
avGFP	Aequorea victoria green fluorescent protein
*dfi*	Differential flexibility index
ESPT	Excited-state proton transfer
PRS	Perturbation response scanning
ENM	Elastic network model
pK_a_^app^	Apparent acid dissociation constant
